# Instillation timing as the main determinant of aqueous humor nepafenac exposure

**DOI:** 10.3389/fmolb.2026.1913752

**Published:** 2026-10-05

**Authors:** Angelo Macri, Annalisa Salis, Margherita Tarallo, Michele Iester, Gianluca Damonte, Francesca Mattioli

**Affiliations:** 1 IRCCS AOM Azienda Ospedaliera Metropolitana - Ospedale Policlinico San Martino, Genoa, Italy; 2 Department of Experimental Medicine (DIMES), University of Genoa, Genoa, Italy; 3 Department of Neuroscience, Rehabilitation, Ophthalmology, Genetics, Maternal and Child Health (DINOGMI), University of Genoa, Genoa, Italy; 4 Department of Internal Medicine (DIMI), Pharmacology and Toxicology Unit, University of Genoa, Genoa, Italy

**Keywords:** amfenac bioactivation, aqueous humor, cataract surgery, instillation timing, nepafenac, ocular pharmacokinetics

## Abstract

**Purpose:**

To evaluate aqueous humor concentrations of nepafenac following topical administration of 0.3% and 0.1% formulations, to assess the influence of timing of instillation on intraocular exposure.

**Methods:**

Aqueous humor samples collected during anterior segment surgery were analyzed. Nepafenac concentrations were quantified in 151 samples (0.3% instilled once daily: n = 73; 0.1% instilled three times daily: n = 78). Amfenac was quantified only in the nepafenac 0.3% group (n = 73), owing to the stepwise validation of the analytical method (see Limitations). Due to right-skewed distributions, concentrations were summarized as median (IQR) and geometric mean with 95% confidence intervals Mann–Whitney U test and Spearman’s rank correlation coefficient were applied. Postoperative optical coherence tomography was performed at 1, 2, and 3 months to assess the occurrence of Irvine–Gass syndrome.

**Results:**

Geometric mean nepafenac concentrations were 22.13 nM (95% CI 15.85–30.91) for 0.3% and 21.73 nM (95% CI 13.29–35.53) for 0.1% (p = 0.319). Within the 0.1% group, instillation timing significantly influenced concentrations (p < 0.001), with markedly higher levels approximately 30–60 min after instillation compared with the 10–12 h subgroup. Amfenac correlated with nepafenac (ρ = 0.56, p < 0.001). No cases of clinically evident Irvine–Gass syndrome were observed.

**Conclusion:**

No cases of clinically evident Irvine–Gass syndrome were observed; however, the study was not powered or designed to assess clinical efficacy. Timing of instillation is a major determinant of aqueous humor nepafenac concentrations. Preoperative instillation shortly before surgery may optimize intraoperative drug availability and support maintenance of mydriasis. Standardized sampling is essential for robust pharmacokinetic comparisons.

## Introduction

Pathologies or trauma affecting the anterior segment of the eye, including minimally invasive ocular surgery, may induce postoperative ocular inflammation of varying severity. Cataract extraction is one of the most commonly performed surgical procedures worldwide and has undergone substantial technological advances over the past 2 decades (Cicinelli et al., 2023). Despite these improvements, postoperative inflammation remains a relevant clinical issue and, if inadequately controlled, may result in ocular discomfort or clinically significant cystoid macular edema (CME), also known as Irvine–Gass syndrome. Although the incidence of CME has decreased with modern phacoemulsification techniques, the release of inflammatory mediators continues to play a central pathogenic role (Tongal et al., 2025; Iftikhar et al., 2023).

According to the European Society of Cataract and Refractive Surgeons (ESCRS) guidelines, the postoperative management of cataract surgery benefits from topical nonsteroidal anti-inflammatory drugs (NSAIDs) administration. The use of these agents has been shown to be effective in reducing postoperative inflammation and in preventing CME following routine cataract surgery (European Society of Cataract and Refractive Surgeons, 2025; Han et al., 2019).

In addition to postoperative administration, several authors have investigated the clinical benefits of initiating topical anti-inflammatory therapy prior to surgery. Evidence from large-scale studies suggests that preoperative prophylactic dosing of topical NSAIDs significantly decreases the incidence of postoperative CME compared to placebo (Ferro Desideri et al., 2025).

NSAIDs exert both anti-inflammatory and analgesic effect, primarily through inhibition of the cyclooxygenase (COX) pathway and subsequent suppression of prostaglandin (PG) synthesis (Bezatis et al., 2022). Cataract surgery induces an increase in intraocular PG levels, leading to disruption of the blood–aqueous barrier, increased vascular permeability, vasodilation, and elevation of intraocular pressure, all of which contribute to postoperative inflammation. Among COX isoforms, COX-2 is recognized as the principal mediator of this inflammatory response and is markedly upregulated in experimental models of ocular injury or inflammation (Russo et al., 2013).

In addition to their role in controlling postoperative inflammation and pain, topical NSAIDs are essential for maintaining intraoperative mydriasis by inhibiting PG-mediated pupil constriction during phacoemulsification (Bucci and Waterbury, 2011).

Several topical NSAIDs are currently available for ophthalmic use, including ketorolac, diclofenac, indomethacin, bromfenac, and nepafenac (Russo et al., 2013).

Among these agents, bromfenac and nepafenac offer the advantage of simplified dosing regimens, which may improve treatment adherence, particularly in elderly patients, while maintaining anti-inflammatory and analgesic efficacy comparable to other NSAIDs. Bromfenac has demonstrated favorable ocular penetration and sustained concentrations in the aqueous humor for up to approximately 12 h after instillation (Macrì et al., 2017).

Its clinical efficacy has been documented in various settings, including postoperative inflammation, allergic conjunctivitis, and as adjunctive therapy in exudative age-related macular degeneration (Macrì et al., 2017; Hoy, 2015).

Nepafenac is a widely used topical NSAID indicated for the management of postoperative inflammation and ocular pain following cataract surgery (Bezatis et al., 2022).

It is a neutral prodrug that readily penetrates the cornea and is subsequently hydrolyzed by intraocular tissues to its active metabolite, amfenac, which inhibits prostaglandin H synthase (Almasri et al., 2025).

Nepafenac received EMA approval in 2007 as a 0.1% (1 mg/mL) ophthalmic suspension administered three times daily for the prevention and treatment of postoperative pain and inflammation following cataract surgery, and for reducing the risk of macular edema in diabetic patients undergoing the procedure (Cable, 2012; Silverstein, 2019).

The efficacy and safety of nepafenac 3 mg/mL (0.3%) administered once daily were further demonstrated in large randomized, double-blind, placebo-controlled clinical trials (Modi et al., 2014).

Whether the once-daily 0.3% regimen achieves aqueous exposure comparable to the three-times-daily 0.1% regimen has clinical relevance for balancing efficacy against treatment adherence.

Assessment of ocular drug disposition following topical administration can be achieved by measuring drug and metabolite concentrations in biological fluids, particularly in the aqueous humor. Numerous human studies have evaluated ocular penetration and pharmacokinetic profiles of ophthalmic antibiotics and NSAIDs to determine whether therapeutic concentrations are achieved in target tissues (Wolf et al., 2007).

The aim of the present study was to evaluate aqueous humor concentrations of nepafenac following topical administration of 0.3% and 0.1% formulations, to assess the influence of timing of instillation on intraocular exposure.

Although aqueous humor concentrations of nepafenac have been previously reported, the influence of the interval between instillation and intraocular sampling on measured drug exposure has not been systematically investigated, and this variable may confound comparisons between formulations and dosing regimens.

The primary endpoint was the aqueous humor concentration of nepafenac and its dependence on the instillation-to-sampling interval. Secondary, exploratory endpoints were amfenac concentration (0.3% group) and the postoperative occurrence of cystoid macular edema assessed by OCT.

## Methods

This prospective, single-center study enrolled volunteers scheduled for phacoemulsification and intraocular lens implantation at the University of Genoa’s Department of Ophthalmology (IRCCS AOM Azienda Ospedaliera Metropolitana - Ospedale Policlinico San Martino, Genoa, Italy) from January 2017 onwards. The protocol received approval from the Liguria Regional Ethics Committee (BROMUA01 Study, Registration number: 126/12; registration number substantial amendments: 015/29.10.2015), and all participants provided written informed consent in accordance with the Declaration of Helsinki.

Inclusion criteria consisted of a cataract diagnosis significant enough to require surgical extraction. Exclusion criteria were a history of prior intraocular surgery, recent ocular trauma or uveitis, severe corneal epithelial damage or other corneal complications as judged by the investigator, use of topical medications known to potentially interfere with the study measurements, severe hepatic impairment, and active oncological treatment. Concomitant systemic medication was recorded at baseline; no patient was receiving regular systemic anti-inflammatory therapy during the study period. All patients underwent fundus examination and optical coherence tomography within 1 week before surgery. Patients with clinically evident diabetic retinopathy or macular edema were excluded.

Eligible subjects were consecutively recruited to receive a single 50 µL drop of nepafenac 0.1% or 0.3% suspension in the eye designated for surgery. Allocation to the 0.1% or 0.3% formulation was based on routine clinical practice at the discretion of the treating surgeon, and was not randomized.

The last administration time was recorded.

At the onset of the surgical procedure, prior to the administration of any intraocular irrigating solution, approximately 0.1–0.2 mL of undiluted aqueous humor was aspirated. This was performed via peripheral clear cornea paracentesis using a 27-gauge needle attached to an insulin microsyringe. Utmost care was taken to prevent blood contamination or mechanical trauma to the iris and surrounding tissues, with aspiration ceasing immediately upon the initial shallowing of the anterior chamber. Samples were transferred to plastic vials and transported in a dedicated dry ice container to the laboratory, where they were stored at −80 °C until analysis.

At the conclusion of the surgery, all patients received a 0.1 mL intracameral injection of cefuroxime sodium as standard prophylaxis.

Aqueous humor samples have been collected and analyzed using high-performance liquid chromatography coupled with electrospray ionization mass spectrometry (HPLC–ESI–MS).

The concentrations of nepafenac and its active metabolite, amfenac, in aqueous humor were simultaneously quantified using high-performance liquid chromatography coupled with electrospray ionization mass spectrometry (HPLC–ESI–MS). The analytical system consisted of an Agilent 1,260 Infinity µ-HPLC unit interfaced with a Thermo Fisher Scientific LCQ Fleet ion-trap mass spectrometer.

Chromatographic separation was carried out on an Agilent Zorbax column (1 × 150 mm, 3.5 μm), maintained at 30 °C, with a flow rate of 40 μL/min. The mobile phase comprised water containing 0.1% formic acid (phase A) and acetonitrile with 0.05% formic acid (phase B). The gradient initiated at 98% phase A for 10 min, followed by a linear shift to 5% phase A over 25 min, held for 5 min, and then returned to initial conditions for column re-equilibration.

A 10-µL aliquot of each sample and standard was injected directly without any pre-extraction. Calibration standards for nepafenac and amfenac were prepared in artificial aqueous humor (Macri et al., 2015). Detection was performed in positive-ion mode using Selected Reaction Monitoring (SRM), employing the specific transition (255 → 338) for both analytes. The method was validated in accordance with standard analytical criteria, including accuracy, precision and stability. The lower limit of quantification (LLOQ) was 0.1 nM for nepafenac and 0.2 nM for amfenac, defined as the concentration yielding a peak signal-to-noise ratio of 10 relative to the blank background. Samples with concentrations below the LLOQ were considered non-quantifiable and excluded from the statistical and pharmacokinetic analyses. Calibration curves were generated in triplicate to ensure robust intra-day performance throughout each analytical batch. During the earlier phase of the study the method was validated for nepafenac only; simultaneous quantification of amfenac was incorporated and validated in a subsequent phase (see Limitations).

As part of the postoperative prophylactic regimen, patients continued the formulation assigned during the preoperative phase for 2 weeks: those in the 0.1% group instilled nepafenac 0.1% eye drops three times daily for 2 weeks, those in the 0.3% group instilled nepafenac 0.3% eye drops once daily for 2 weeks.

No other topical anti-inflammatory therapy (NSAIDs or corticosteroids) was administered during the preoperative or postoperative period, apart from the intracameral cefuroxime prophylaxis described above.

Postoperative optical coherence tomography (OCT) evaluation was performed at 1, 2, and 3 months after surgery to assess the onset of cystoid macular edema (Irvine–Gass syndrome).

### Statistical analysis

Due to right-skewed distributions, concentrations were summarized using median (interquartile range) and geometric mean (95% CI). Between-group comparisons were performed using the two-sided Mann–Whitney U test. Within the 0.1% group, two timing subgroups were defined *post hoc* on the basis of the recorded administration-to-sampling interval, corresponding to clinically meaningful pharmacokinetic windows: an early subgroup sampled 30–60 min after instillation and a trough subgroup sampled 10–12 h after instillation, immediately before the next scheduled dose. These subgroups were compared using the two-sided Mann-Whitney U test. Correlations were assessed using Spearman’s rank correlation coefficient.

## Results

A total of 151 aqueous humor samples collected during anterior segment surgery were analyzed (0.3% instilled once daily: n = 73; 0.1% instilled three times daily: n = 78). The demographic characteristics of the study population are summarized in Table 1.

**TABLE 1:** Patients’ demographics.

Group	N Subjects	Male, n	Female, n	Age (mean ± SD), years
Total	151	54	97	73 ± 7
Nepafenac 0.3%	73	25	48	71 ± 6
Nepafenac 0.1%	78	29	49	74 ± 8

In the 0.3% cohort, the mean (SD) interval between instillation and aqueous humor sampling was 11.52 ± 1.69 h (median 11.50 h), whereas in the 0.1% cohort the mean (SD) interval was 8.76 ± 5.09 h (median 11.13 h). Amfenac was quantified in 73 samples (nepafenac 0.3% group).

Geometric mean nepafenac concentrations were 22.13 nM (95% CI 15.85–30.91) for the 0.3% formulation and 21.73 nM (95% CI 13.29–35.53) for the 0.1% formulation, with no statistically significant difference between groups (p = 0.319). However, within the 0.1% cohort, instillation timing significantly influenced drug levels (p < 0.001). Samples collected 30–60 min after instillation demonstrated markedly higher concentrations compared with the 10–12 h subgroup (adjusted p < 0.001). Mean concentrations of nepafenac and amfenac according to formulation and timing subgroup are reported in Table 2.

**TABLE 2:** Nepafenac and amfenac aqueous humor concentrations according to formulation and timing of instillation.

Group	Nepafenac geometric mean (95% CI), nM	Nepafenac median (IQR), nM	Amfenac geometric mean (95% CI), nM	Amfenac median (IQR), nM
Nepafenac 0.3% (10–12 h)	22.13 nM, 95% CI 15.85–30.91	19.02 (7.72–67.93)	57.66 (45.88–72.47)	63.27 (33.86–133.14)
Nepafenac 0.1% (10–12 h)	7.47 (5.33–10.47)	6.53 (4.05–13.59)	—	—
Nepafenac 0.1% (30–60 min)	627.38 (402.43–978.08)	707.30 (521.85–1,200.41)	—	—

In the 0.3% group, amfenac concentrations are reported as geometric mean (95% CI) and median (IQR) in Table 2, and correlated positively with nepafenac (ρ = 0.56, p < 0.001), confirming intraocular bioactivation of the prodrug.

The distribution of nepafenac concentrations according to formulation and timing is illustrated in Figure 1, which shows the wide inter-patient variability and the right-skewed distribution of measured concentrations.

**FIGURE 1:**
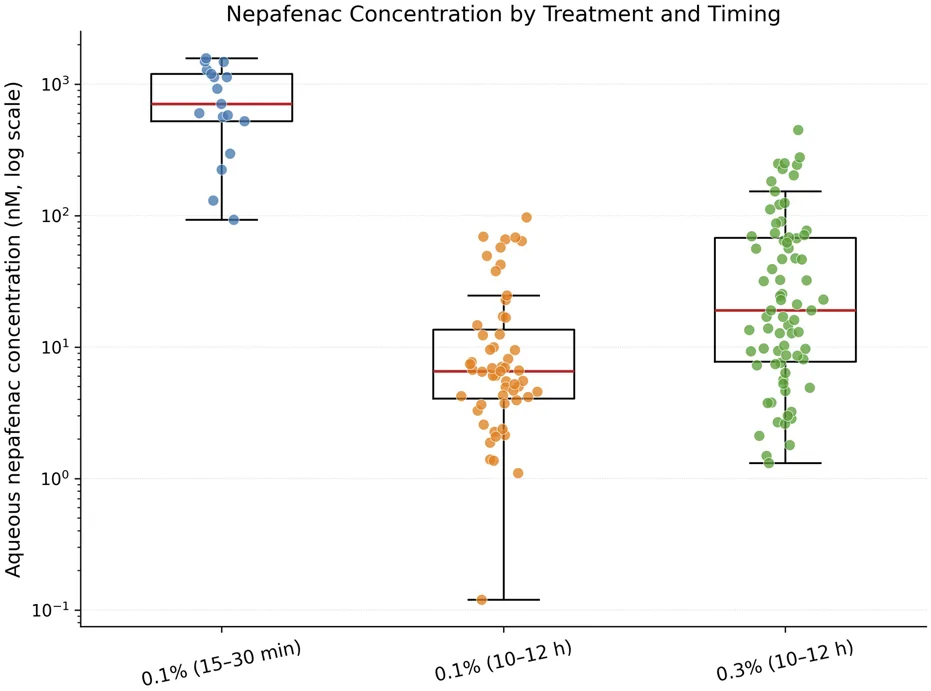
Distribution of nepafenac aqueous humor concentrations (log scale) according to formulation and timing of instillation. Boxplots represent median and interquartile range with individual data points.

## Discussion

The present study demonstrates that the primary determinant of aqueous humor nepafenac concentration is the timing of instillation. Samples collected approximately 30–60 min after administration showed markedly higher concentrations compared with those obtained at longer post-dose intervals, consistent with peak intraocular penetration. These findings underscore the importance of standardized sampling protocols when evaluating ocular pharmacokinetics and when comparing different dosing regimens or formulations.

Inter-patient variability in the interval between the last instillation and aqueous sampling was substantial and likely contributed to the observed dispersion in concentration values. The markedly larger standard deviation in the 0.1% group reflects a wide range of sampling times, including early post-dose collections corresponding to the highest concentration outliers. This temporal heterogeneity explains the pronounced right-skewed distributions observed and the discrepancy between arithmetic means and medians, further confirming that administration-to-sampling timing is a major driver of measured aqueous concentrations. The similar overall geometric means between the two formulations should not be interpreted as evidence of equivalence, since the wide and unbalanced distribution of sampling times—especially in the 0.1% arm—may itself drive this apparent similarity.

A methodological strength of the present investigation is the use of artificial aqueous humor as calibration and reference matrix for chromatographic quantification. Unlike calibration performed in simple solvent systems, a matrix mimicking the physicochemical characteristics of human aqueous humor minimizes matrix effects and enhances analytical reproducibility at low concentration ranges. This approach, previously described and validated by our group (Macri et al., 2015), improves the reliability of quantitative measurements and facilitates inter-study comparability of anterior chamber drug concentrations. Given the limited availability of true blank human aqueous humor for calibration purposes, the use of an artificial matrix represents a scientifically robust and practical alternative in ocular pharmacokinetic research.

For contextual interpretation, our findings may be compared with previously published data from our group on bromfenac 0.09% ophthalmic solution (Macrì et al., 2017). In that study, the mean aqueous humor concentration of bromfenac was 37.60 ± 68.86 nM, demonstrating measurable and sustained anterior chamber penetration. Importantly, these concentrations exceeded the reported COX-2 IC_50_ value of approximately 7.5 nM, corresponding to levels roughly fivefold higher than the enzymatic inhibitory threshold. This pharmacokinetic-pharmacodynamic relationship supported sustained COX-2 inhibition within the anterior chamber. This comparison should be interpreted with caution, as the bromfenac value represents an arithmetic mean from our previous study, whereas nepafenac concentrations in the present cohort are reported as geometric means; the marked right-skew and differing summary statistics limit a direct numerical comparison between the two agents.

Nepafenac, however, is a neutral prodrug that undergoes rapid intraocular bioactivation to its active metabolite, amfenac. Preclinical enzymatic studies have demonstrated that amfenac inhibits both COX-1 (IC_50_ ≈ 0.25 µM) and COX-2 (IC_50_ ≈ 0.15 µM), indicating potent but relatively non-selective cyclooxygenase inhibition. These values, reported by Gamache et al. (2000) and cited in the EMA regulatory assessment document for nepafenac, European Medicines Agency (2026) correspond to approximately 250 nM and 150 nM, respectively. When compared on a purely arithmetic basis, the geometric mean aqueous nepafenac concentrations measured in our cohort (∼22 nM) are below the nominal *in vitro* IC_50_ values reported for amfenac, corresponding to approximately 0.15× the COX-2 IC_50_.

However, interpretation of such concentration-to-IC_50_ ratios requires caution. As highlighted in both preclinical literature and the EMA scientific assessment, nepafenac exhibits minimal direct COX inhibitory activity as a parent compound and relies on intraocular conversion to amfenac for pharmacological effect. Aqueous humor concentrations of nepafenac may therefore underestimate intracellular active metabolite levels at the site of inflammation. Furthermore, IC_50_ values derived from isolated enzyme systems do not fully account for tissue distribution, protein binding, repeated dosing, or cumulative prostaglandin suppression *in vivo*. Accordingly, while bromfenac demonstrates greater intrinsic enzymatic potency and achieves aqueous concentrations exceeding its reported IC_50_, the present pharmacokinetic data do not permit conclusions regarding comparative clinical superiority. Both agents achieve measurable intraocular exposure and remain established therapeutic options in the prevention of postoperative ocular inflammation.

No cases of postoperative cystoid macular edema were observed during follow-up, consistent with the recognized anti-inflammatory efficacy of topical NSAIDs. Although this study was not designed to evaluate clinical outcomes, this is a descriptive observation, as the study was not powered for clinical endpoints. The pharmacokinetic data support the rationale for preoperative administration shortly before surgery in order to maximize intraocular exposure during the peak inflammatory stimulus associated with phacoemulsification. Prostaglandins released in response to surgical trauma induce iris sphincter contraction and intraoperative miosis; by achieving sufficient anterior-chamber concentrations at the time of surgery, nepafenac—through its active metabolite amfenac—suppresses this prostaglandin-mediated response and helps preserve mydriasis.

Several limitations should be acknowledged. First, allocation to the two formulations was not randomized, which may introduce selection bias; this should be considered when interpreting between-group comparisons. Second, amfenac quantification was not available for all patients and was not performed in the 0.1% treatment group. This reflects the stepwise implementation and validation of the analytical method during the study period, whereby reliable and fully validated quantification of the active metabolite was achieved during the later phase of recruitment. In the earlier phase, the assay had been validated for the parent compound nepafenac only, and the 0.1% samples were analyzed under this single-analyte method. Because aqueous-humor volumes are minimal (0.1–0.2 mL) and samples were injected directly without pre-extraction, these specimens were no longer available for retrospective re-assay once the validated amfenac method became available. Consequently, direct comparison of metabolite exposure between dosing regimens was not possible, and therefore no conclusions can be drawn regarding differential bioactivation between formulations. Importantly, this limitation does not affect the principal finding, which is based on nepafenac measured uniformly across both groups; amfenac quantification served only to confirm intraocular bioactivation of the prodrug within the 0.3% subset. Uniform quantification of both parent compound and active metabolite across all treatment groups, ideally at standardized sampling times, would allow a more complete pharmacokinetic–pharmacodynamic characterization, including formulation-specific bioactivation efficiency.

Third, variability in the interval between last instillation and aqueous sampling, particularly in the 0.1% cohort, introduces heterogeneity when interpreting overall concentration averages. Although timing sub-analyses were performed, future pharmacokinetic investigations would benefit from strict standardization of preoperative dosing schedules or inclusion of administration-to-sampling interval as a formal covariate in statistical models.

Finally, aqueous humor concentrations may not fully reflect drug distribution within intraocular tissues, especially posterior segment structures that may also contribute to clinical efficacy in preventing postoperative macular edema.

## Conclusion

Instillation timing significantly affects aqueous humor nepafenac concentrations and may confound dose comparisons. Standardized sampling protocols are therefore essential for reliable pharmacokinetic evaluation of topical ophthalmic NSAIDs.

Our pharmacokinetic data suggest, but do not directly demonstrate, that preoperative administration shortly before surgery may optimize intraocular drug exposure during the peak inflammatory stimulus and support maintenance of intraoperative mydriasis.

As a hypothesis-generating proposal, instillation shortly before surgery may correspond to the peak intraocular exposure observed in this study. This proposal requires confirmation in prospective clinical outcome studies before any change in dosing recommendations can be made.

## Data Availability

The raw data supporting the conclusions of this article will be made available by the authors, without undue reservation.
